# A comparative analysis of carer-employees in Canada over time: a cross-sectional analysis of Canada’s General Social Survey, 2012 and 2018

**DOI:** 10.17269/s41997-023-00762-9

**Published:** 2023-05-10

**Authors:** Jerry Wu, Allison Williams, Li Wang, Peter Kitchen

**Affiliations:** 1 Department of Mathematics, University of Waterloo, Waterloo, ON Canada; 2 School of Earth, Environment & Society, McMaster University, Hamilton, ON Canada; 3 Offord Center for Child Study & Department of Psychiatry & Behavioural Neurosciences, McMaster University, 1280 Main Street West, Hamilton, ON L8S 4K1 Canada

**Keywords:** Carer-employees, GSS, Work-related, Health, Life satisfaction, Work-life balance, Employés soignants, ESG, lié au travail, santé, satisfaction à l’égard de la vie, équilibre entre travail et vie personnelle

## Abstract

**Objectives:**

The aims of the study are to identify trends in the socio-demographic, health, and work profiles of Canadian carer-employees (CEs) over time, as well as the gender difference in the intensity of caring.

**Methods:**

Cross-sectional data from cycles 26 and 32, collected in 2012 and 2018 respectively, of the Canadian General Social Survey (GSS) were used. Logistic, multinomial logistic, and linear regressions were used to estimate how caregiving is associated with caregivers’ health, well-being, and work in both cycles. Regressions from both cycles were then compared with chi-square tests for significant differences over time.

**Results:**

The proportion of male CEs grew between 2012 and 2018, and women were no longer more likely to be a CE. The intensity of care for female CEs was significantly increased from 2012 to 2018 as compared with their male counterparts. General health (2018: OR = 0.25[0.11, 0.61] vs. 2012: OR = 0.33[0.15, 0.72]) and life satisfaction ($\beta$ = -0.42[0.54, -0.30] vs. $\beta$ = -0.22[-0.30, -0.14]) were significantly worsened with respect to the role of CEs from 2012 to 2018.

**Conclusion:**

Our study provides the evidence that CEs’ health and well-being have worsened over time, especially for female CEs, indicating that the needs of CEs are growing at a faster rate than the supports available. The results are meaningful in informing and justifying the provision of CE supports at work in order to sustain CEs in the workplace, such as the carer-friendly workplace policies.

## Résumé

**Objectifs:**

Notre étude vise à cerner les tendances du profil sociodémographique, sanitaire et professionnel des employés soignants (ES) canadiens au fil du temps, ainsi que les différences entre les sexes dans l’intensité des soins.

**Méthode:**

Nous avons utilisé les données transversales des cycles 26 et 32, collectées en 2012 et en 2018 respectivement, de l’Enquête sociale générale (ESG) du Canada. Nous avons fait appel à la régression logistique, à la régression logistique multinomiale et à la régression linéaire pour estimer les associations entre la prestation de soins et la santé, le bien-être et le travail des soignants lors des deux cycles. Nous avons ensuite comparé les régressions des deux cycles avec les analyses du khi-carré pour cerner les écarts significatifs dans le temps.

**Résultats:**

La proportion d’ES de sexe masculin a augmenté entre 2012 et 2018, et les femmes n’affichaient plus une probabilité accrue d’être des ES. L’intensité des soins chez les ES de sexe féminin a augmenté de façon significative entre 2012 et 2018 comparativement à leurs homologues masculins. La santé générale (2018 : RC = 0,25[0,11, 0,61] vs 2012 : RC = 0,33[0,15, 0,72]) et la satisfaction à l’égard de la vie ($\beta$ = -0,42[0,54, -0,30] vs $\beta$ = -0,22[-0,30, -0,14]) se sont significativement dégradées en ce qui a trait au rôle des ES entre 2012 et 2018.

**Conclusion:**

Notre étude prouve que la santé et le bien-être des ES se sont dégradées au fil du temps, surtout chez les femmes, ce qui montre que les besoins des ES augmentent plus rapidement que le soutien disponible. Ces résultats contribueront à éclairer et à justifier la prestation de mesures d’aide aux ES afin de les appuyer dans leurs milieux de travail, comme par exemple des politiques conçues pour les soignants en milieu de travail.

## Introduction

In 2018, approximately one in four Canadians aged 15 and older (or 7.8 million people) provided informal care to family and friends, coinciding with the phenomenon of an increasingly aged population (Hango, 2020). Employment and Social Development Canada (2014) projects that by 2030, people aged 65 and over will make up 23% of Canadians, compared to 15.6% in 2014. As the demand for caregiving grows, the number of carer-employees (CEs) simultaneously grows. CEs are those who work in paid employment while still providing informal care to adult family or friends (Ireson et al., 2018). In 2012, CEs accounted for approximately 35% of the workforce, or roughly 5.6 million Canadian employees (Wang et al., 2018), and made up the majority of unpaid carers in Canada. CEs often have a range of care responsibilities, including managing doctor appointments, providing emotional support, providing transportation, and assisting with mobility.

Studies show carers typically have worse mental and physical health compared to the general population (Ramesh et al., 2017). Research has revealed that unpaid caring is associated with “stress, fatigue, anxiety, depression, sleep loss, muscle pain, and other conditions” (Etters et al., 2008; Williams et al., 2016). Carers can find caregiving to be stressful, time-consuming, and emotionally draining (Sherman, 2018), and the intensity of unpaid caring often leads to unfavourable moods and depression (Sethi et al., 2017). The trend across time seems to indicate that, for carers, negative emotions of distress, depression, anger, and the feeling of being helpless in the ability to continue with caring responsibilities are worsening; between 2009 and 2010, 15.6% felt this way, and between 2013 and 2014, this figure increased to 33.3% (Health Quality Ontario, 2017).

This paper is specifically interested in CEs due to the double role they occupy, and the stresses that are uniquely associated with the intersection between paid work and unpaid caregiving. For CEs, balancing work and family is an area of difficulty and a source of stress. Health, well-being, and workplace productivity can be negatively impacted (Halinski et al., 2020; Kim & Schulz, 2008). Especially for women, caregiving negatively affects work outcomes (Williams et al., 2017). The way CEs choose to handle imbalances in job and home life often means forgoing personal time. Research shows that CEs are more likely to handle issues related to work and family by sacrificing personal time rather than lowering their workplace productivity (Allen et al., 2014; Halinski et al., 2020). This can be particularly harmful since caregiving responsibilities often lead CEs to relegate paid work to outside the regular workweek; this may cause them to abort hobbies, social activities, and vacations, which result in negative health outcomes (Wang et al., 2018). Due to a combination of these factors, the stress of managing two demanding roles can lead CEs to make the decision to permanently leave the labour force (Wang et al., 2018).

To better gauge the general trends in unpaid caring with respect to the caregiver’s health, well-being and work-life balance in Canada, four principal research questions will be examined herein:

1. Using the latest Canadian General Social Survey (GSS) 2018, what is the current socio-demographic, health, well-being, and work-related profile of CEs? Are there any significant differences as compared to 2012?
2. Are the CEs more likely to have poorer health, well-being and work-related outcomes compared to the non-CEs, controlling for a range of possible confounders? Does the association become worse or better in 2018 as compared to 2012?
3. Are there significant gender differences among CEs in the general health, well-being and work-related outcomes, controlling for a range of possible confounders? Do the gender differences change between 2012 and 2018?
4. Do the female CEs do more time-intensive tasks than male CEs with respect to the total number of hours caring per week and the frequency of providing care or help with transportation, household chores, house maintenance, medical treatment, scheduling and banking? Are there any significant differences between 2012 and 2018?

## Methods

### Data source

We used data from the 2012 and 2018 cycles of Canada’s General Social Survey (GSS), a nation-wide cross-sectional survey that collects information on Canadians who provide care to family and friends with a long-term health condition, physical or mental disability or problems related to aging, as well as individuals who receive this care and about the challenges both groups face. The sampling frame combines landline and cellular telephone numbers from the Census and various administrative sources with Statistics Canada’s dwelling frame (Statistics Canada, 2018a). The overall response rate is 65.7% for 2012 GSS and 52.8% for 2018 GSS.

The 2018 survey included 20,180 respondents aged 15 years and older, while the 2012 survey included 23,025. The target population selected for this study was employees. In the 2018 sample, employees were defined as people who either answered yes to “Last week, did you work at a job or business?” or “Last week, did you have a job or business from which you were absent?” with the reason for absence not being “Temporary layoff due to business conditions”, “Seasonal layoff”, or “You had a casual job and no work was available”; or answered yes to “In the past 12 months, did you work at a job or business?”. Since different questions were asked in the 2012 survey, employees were defined as people who identified their main activity during the past 12 months as working at a paid job or business; answered yes to “Were you employed or self-employed at any time last week?”; or answered yes to “Were you employed or self-employed at any time during the past 12 months?”. In total, the 2018 survey included 12,368 employees and the 2012 survey included 13,860 employees.

### Caregiver status

In both surveys, CEs were defined as employees who answered yes to “During the past 12 months, have you helped or cared for someone who had a long-term health condition or a physical or mental disability?” or “During the past 12 months, have you helped or cared for someone who had problems related to aging?”. Since studies show carers of young children (who are most often young parents) and carers of older adults (who are usually spouses or children of the care-receiver) differ in areas of well-being, such as carer burden and mental health (de Oliveira et al., 2015), CEs were also limited to carers whose primary care-receivers were 18 years of age or older.

### Dependent variables

#### Health, well-being and work-related outcomes

Several variables related to health, well-being, and work were explored in relation to caregiving. General health and mental health were dichotomized into good (good, very good, excellent) and poor (poor, fair). Daily stress was collapsed into three categories: low (not at all stressful, not very stressful), medium (a bit stressful), and high (quite a bit stressful, extremely stressful).

Adapted from the APA Dictionary of Psychology, stress is a normal reaction to everyday pressures. Over time, the accumulation of everyday stress responses can erode our sense of well-being and lead to low mood and a feeling of being on edge all the time. That is when stress becomes distress, often involving negative affect and physiological reactivity. Difficulty balancing job/home life was constructed by combining the responses to two separate questions: “In the past 12 months, how often has it been difficult to concentrate or fulfill your work responsibilities because of your family responsibilities?” and “In the past 12 months, how often has it been difficult to fulfill family responsibilities because of the amount of time you spent on your job?”. The 2018 sample uses a 5-point Likert scale (never, rarely, sometimes, often, always) while the 2012 sample uses a 4-point Likert scale (never, sometimes, often, always) to gauge responses, hence, we collapsed the “never” and “rarely” categories from the 2018 survey. The scores from the responses were added and three categories were created: low difficulty (2–3), medium difficulty (4–6), and high difficulty (7–8). Work-life balance satisfaction was also categorized into three categories: satisfied (very satisfied, satisfied), indifferent (neither satisfied nor dissatisfied), and dissatisfied (dissatisfied, very dissatisfied). Finally, life satisfaction was kept on a scale from 1 to 10.

#### Intensity of care

Among the CEs, we examined the gender differences in the intensity of care measured by the number of hours’ care or help provided per average week. We also checked the frequency of providing help or care for the following specific caregiving tasks: (1) transportation to do shopping, run errands, or get to medical appointments or social events, (2) household chores, such as meal preparation, meal clean-up, house cleaning, laundry or sewing, (3) house maintenance or outdoor work, (4) medical treatments or procedures, (5) scheduling or coordinating care-related tasks, and (6) banking, bill paying or managing finances. We focused on the gender difference in the daily care and the care at least once a week for each activity.

### Socio-demographic variables

A selection of variables were used both to evaluate the socio-demographic profile of CEs and as control variables in determining the relationships between caregiving and health, well-being, and work-related outcomes. These included age, sex, marital status, hours worked a week, landed immigrant status, urban–rural classification, education level, family income, and occupation.

The occupation was defined by the National Occupational Classification (NOC) (Government of Canada, 2021) and synthesized into 6 groups based on the characteristics of the work (Table 1). All variables and categories are displayed in Table 2.

**Table 1:** Description of occupation group used in the analysis

Occupation group name	Description
Management	Management occupations
Business	Business, finance, administration occupations; Sales and service occupations
Hard sciences	Natural and applied sciences and related occupations
Health	Health occupations
Socially-related	Occupations in education, law and social science, community, government; Occupations in art, culture, recreation and sport
Manual work	Occupations in trades, transport, equipment operators; Occupations in natural resources, agriculture; Occupations in manufacturing and utilities

**Table 2:** Summary statistics of socioeconomics variables by caregiver status, GSS cycles 2012 and 2018

	GSS cycle 2018			GSS cycle 2012		
	CE	Non-CE	Total	CE	Non-CE	Total
n	2675	9693	12,368	3711	10,149	13,860
Sex, %						
Female	50.04	46.91	47.59	50.89	45.27	46.77
Male	49.96	53.09	52.41	49.11	54.73	53.23
Marital Status, %						
Married	65.43	62.84	63.4	66.41	63.99	64.63
Formerly married	8.15	6.36	6.75	6.65	6.71	6.7
Single	26.41	30.8	29.85	26.95	29.3	28.67
Education, %						
High school diploma and below	32.1	34.17	33.73	37.99	38.26	38.19
Trades, college, CEGEP, and other	29.82	29.78	29.79	30.44	29.48	29.74
Bachelor’s degree or lower university degree	27.02	25.74	26.02	23.13	23.6	23.48
University degree above bachelor’s	11.06	10.31	10.47	8.44	8.65	8.6
Immigrant, %						
Non-immigrant	80.8	76.09	77.11	85.74	78.61	80.53
Immigrant	19.2	23.91	22.89	14.26	21.39	19.47
Urban/Rural, %						
Rural	15.7	14.9	15.07	18.91	16.74	17.32
Urban	84.3	85.1	84.93	81.09	83.26	82.68
Income Groups, %						
< $40 K	12.09	13.94	13.54	11.82	13.09	12.74
$40–$60 K	11.21	11.85	11.71	13.22	14.75	14.33
$60–$80 K	11.03	12.67	12.31	14.3	15.37	15.08
$80–$100 K	10.35	11.8	11.48	14.02	14.39	14.29
$100 K +	55.33	49.74	50.95	46.64	42.39	43.57
Occupation Group, %						
Management	9.13	8.04	8.27	9.21	8.68	8.82
Business	38.68	42.06	41.33	42.67	43.07	42.96
Hard sciences	8.42	9.71	9.43	6.3	7.38	7.09
Health	8.42	6.87	7.2	6.82	5.41	5.78
Socially-related	18.86	15.06	15.87	13.44	14.74	13.78
Manual work	16.5	18.27	17.89	22.03	20.26	21.55
Age, M (SE)	44 (0.22)	40 (0.16)	41 (0.13)	42 (0.19)	40 (0.16)	40 (0.12)
Hours worked per week, M (SE)	35.25 (0.21)	35.59 (0.15)	35.51 (0.12)	37.16 (0.21)	36.99 (0.16)	37.04 (0.13)

### Analysis

Sampling weights were applied to generate the estimation representing the target population of Canadian employees. The descriptive statistics of two surveys were outlined in total and by caregiver status. We applied logistic regressions to find determinants of caregiving status with respect to a selection of socio-demographic variables using GSS 2012 and 2018 separately. Hausman test was used to examine whether the effect of each variable changed from 2012 to 2018.

Linear regressions were used to examine the gender difference in the intensity of care and multinomial logistic regressions were used to examine the gender gap in the frequency of the specific caring tasks using the sample of CEs.

Logistic regressions were also applied to assess the relationships between CE status and good general and mental health respectively. Multinomial logistic regressions were used to examine the association between CE status and the nature of the stress, difficulty balancing job/home life, and work-life balance satisfaction. Finally, a linear regression was used to model life satisfaction, which used a 10-point scale. All models were run in parallel using GSS 2012 and 2018 separately. Hausman test was used to examine whether the association changed from 2012 to 2018.

Bootstrap method was used to account for the complex survey design. A p-value of less than 0.05 is regarded as significant. All analyses were conducted using STATA 17.0 (StataCorp, College Station, TX, USA, 2021).

## Results

### Descriptive statistics: employees

Our target group was employees, which accounted for 60% of respondents in the 2012 cycle and 61% in the 2018 cycle. Table 2 presents characteristics of employees in total and by caregiver status in both surveys. The profiles of employees were consistent across the two cycles: the majority of them were married, had education above a high school diploma, were non-immigrants, had a family income greater than $100 K, and were most prevalent in business occupation.

The notable changes were found with regard to health and well-being between GSS 2012 and GSS 2018 (see Table 3). There is an increase in poor general health (from 7% to 9%), poor mental health (from 5% to 10%) and high level of stress (from 22% to 24%). While employees reported that both low and high levels of difficulty balancing job/home life increased (from 63% to 68% and from 1.5% to 2.5%, respectively), they reported a decrease at the medium level (from 36% to 30%). The number of employees who reported work-life balance dissatisfaction increased (from 8% to 10%).

**Table 3:** Summary statistics of intensity of care, health and well-being by caregiver status, GSS cycles 2012 and 2018

	GSS cycle 2018			GSS cycle 2012		
	CE	Non-CE	Total	CE	Non-CE	Total
n	2675	9693	12,368	3711	10,149	13,860
Self-reported health, %						
Poor/fair	13.68	7.21	8.61	8.65	6.06	6.76
Excellent/Very good/Good	86.32	92.79	91.39	91.35	93.94	93.24
Self-reported mental health, %						
Poor/fair	14.79	8.94	10.21	6.85	3.95	4.73
Excellent/Very good/Good	85.21	91.06	89.79	93.15	96.05	95.27
Self-reported stress, %						
Low	24.24	30.63	29.24	28.41	32.25	31.22
Medium	49.97	46	46.86	46.49	46.6	46.57
High	25.8	23.37	23.9	25.11	21.15	22.21
Difficulty balancing job/home life, %						
Low	58.54	70.54	67.93	54.62	65.8	62.77
Medium	37.91	27.27	29.58	43.8	32.7	35.72
High	3.54	2.19	2.49	1.58	1.49	1.52
Work-life balance satisfaction, %						
Satisfied	60.61	68.91	67.11	74.4	80.18	78.62
Indifferent	25.68	21.86	22.69	15.46	12.47	13.28
Dissatisfied	13.7	9.23	10.2	10.14	7.35	8.1
Life satisfaction, M (SE)	7.20 (0.03)	7.63 (0.02)	7.53 (0.02)	7.82 (0.02)	8.02 (0.02)	7.96 (0.01)

### Descriptive statistics: carer-employees

In Table 2, the two surveys exhibit mostly consistent results with respect to the profile of CEs: most are women, older, married, non-immigrant and have higher family income compared with non-CEs. In Table 3, in comparison with non-CEs, CEs more frequently reported poor general health (GSS 2012: 9% vs 6%; GSS 2018: 14% vs 7%) and mental health (GSS 2012: 7% vs 4%; GSS 2018: 15% vs 9%) and a high level of stress (GSS 2012: 25% vs 21%; GSS 2018: 26% vs 23%). CEs also were more likely than non-CEs to be dissatisfied with the balance of job and home (GSS 2012: 10% vs 7%; GSS 2018: 14% vs 9%).

With respect to the intensity of care, CEs spent more hours in caring or help per week on average in 2018 (10.3) than in 2012 (7.6). In addition, there was a dramatic increase in the percentages of CEs who provided daily caring in transportation in 2018 as compared with 2012 (8.4% vs 5%), household chores (18.3% vs 14.2%) and scheduling (4.1% vs 1.5%), as shown in Table 4.

**Table 4:** Frequency of caring tasks, GSS cycles 2012 and 2018

Frequency of care	Transportation (%)		Household chores (%)		House maintenance (%)		Medical treatments (%)		Scheduling (%)		Banking (%)	
	2018	2012	2018	2012	2018	2012	2018	2012	2018	2012	2018	2012
No intensive care	61.6	63.3	61.9	67.1	76.4	77.2	86.7	88.3	82.9	90.8	87.2	90.3
At least once a week	30.0	31.7	19.8	18.7	17.7	17.1	7.1	6.4	13.0	7.7	10.2	8.4
Daily care	8.4	5.0	18.3	14.2	5.9	5.7	6.2	5.3	4.1	1.5	2.6	1.4

### Logistic regression on caregiving status

Table 5 reports the results of logistic regression on caregiver status. In both cycles of GSS, those who were older or single (using married/common-law as reference) were more likely to be a caregiver, while immigrants were less likely to be caregivers. However, Hausman test shows the magnitude of immigrants is significantly increased between 2012 and 2018 (Chi-2 = 4.08, *p* < 0.05). Females were significantly more likely to be caregivers in the 2012 model but not in the 2018 model (Chi-2 = 3.71, *p* < 0.05).

**Table 5:** Logistic regression on caregiver status, GSS cycles 2012 and 2018

	GSS cycle 2018		GSS cycle 2012	
	Odds ratio	95% CI	Odds ratio	95% CI
Age	1.027***	**[1.022, 1.031]**	1.019***	**[1.015, 1.023]**
Male (ref. = female)	**0.933**	**[0.804, 1.081]**	0.775***	**[0.689, 0.871]**
Marital Status (ref. = married/common-law)				
Formerly married	**1.146**	**[0.95, 1.382]**	0.876**	**[0.749, 1.024]**
Single	1.35***	[1.119, 1.627]	1.201***	[1.028, 1.403]
Hours Worked	0.998	[0.993, 1.003]	1.002	[0.998, 1.006]
Immigrant (ref. = Canadian born)	0.766***	**[0.644, 0.911]**	0.605***	**[0.52, 0.703]**
Urban (ref. = rural)	1.007	[0.86, 1.179]	0.967	[0.851, 1.1]
Education (ref. = less than high school)				
Trades, college, CEGEP, and other	0.965	[0.826, 1.128]	0.969	[0.851, 1.104]
Bachelor’s degree or lower university degree	1.03	[0.855, 1.241]	0.855**	[0.735, 0.994]
University degree above bachelor’s	0.993	[0.802, 1.229]	0.923	[0.753, 1.132]
Income Groups (ref. = $100 K +)				
< $40 K	0.754**	[0.602, 0.944]	0.792***	[0.666, 0.943]
$40–$60 K	0.777**	[0.636, 0.949]	0.784***	[0.666, 0.923]
$60–$80 K	0.771**	[0.634, 0.939]	0.827**	[0.708, 0.965]
$80–$100 K	0.786**	[0.644, 0.958]	0.881	[0.752, 1.033]
Occupation				
Management	0.852	[0.679, 1.069]	0.969	[0.781, 1.202]
Business	0.779***	[0.65, 0.934]	0.948	[0.802, 1.119]
Hard sciences	0.768*	[0.587, 1.005]	0.854	[0.673, 1.085]
Health	0.96	[0.751, 1.226]	1.112	[0.884, 1.399]
Socially-related	0.791*	[0.625, 1.001]	0.94	[0.764, 1.157]

Bold represents significant difference at 5% level between 2012 and 2018; *p < 0.1; **p < 0.05; ***p < 0.01

### Logistic and linear regressions on health, well-being and work-related variables

For both 2012 and 2018 GSS cycles, caregiving was negatively associated (*p* < 0.01) with good general (GSS 2012: OR = 0.33; GSS 2018: OR = 0.25; Table 6) and mental health (GSS 2012: OR = 0.47; GSS 2018: OR = 0.38; Table 6). Female CEs were more likely to have poor general health in 2012 (GSS 2012: OR = 5.10) than male CEs but the magnitude would decrease with age (GSS 2012: OR = 0.97), however, the association was not significant in 2018. Conversely, regarding the general mental health, the relationship was significant in 2018 but not in 2012 and the magnitude would decrease with age (GSS 2018: OR = 0.98).

**Table 6:** Logistic regression on self-reported general health and mental health, GSS cycles 2012 and 2018

	GSS cycle 2018		GSS cycle 2012	
	Odds ratio	95% CI	Odds ratio	95% CI
**A: Self-Reported General Health**				
Caregiving Status	0.25***	**[0.11, 0.61]**	0.33**	[0.15, 0.72]
Caregiving Status*Male	1.44	[0.48, 4.29]	5.1***	[1.73, 15.03]
Caregiving Status*Age	1.01	[0.99, 1.03]	1.02*	[1, 1.03]
Caregiving Status*Male*Age	1	[0.98, 1.02]	0.97***	[0.95, 0.99]
Age	0.98***	[0.97, 0.98]	0.98***	[0.97, 0.99]
Male (ref. = female)	0.94	[0.73, 1.22]	1.28*	[0.98, 1.67]
Marital Status (ref. = married/common-law)				
Formerly married	0.93	[0.71, 1.2]	0.97	[0.76, 1.25]
Single	0.98	[0.72, 1.33]	0.94	[0.71, 1.24]
Hours Worked	1	[1, 1.01]	1	[0.99, 1]
Immigrant (ref. = Canadian born)	0.63***	[0.51, 0.77]	0.85	[0.67, 1.08]
Urban (ref. = rural)	0.87	[0.69, 1.1]	0.82	[0.65, 1.04]
Education (ref. = less than high school)				
Trades, college, CEGEP, and other	0.94	[0.74, 1.19]	1.1	[0.87, 1.38]
Bachelor’s degree or lower university degree	1.17	[0.89, 1.54]	1.24	[0.94, 1.65]
University degree above bachelor’s	1.05	[0.75, 1.46]	1.33	[0.89, 1.98]
Income Groups (ref. = $100 K +)				
< $40 K	0.47***	[0.36, 0.63]	0.39***	[0.29, 0.53]
$40–$60 K	0.63***	[0.47, 0.83]	0.61***	[0.45, 0.83]
$60–$80 K	0.8	[0.59, 1.09]	0.76*	[0.55, 1.04]
$80–$100 K	0.63***	[0.46, 0.86]	0.58***	[0.43, 0.8]
Occupation				
Management	1.05	[0.73, 1.52]	1.4	[0.88, 2.21]
Business	0.9	[0.68, 1.18]	0.79	[0.59, 1.08]
Hard sciences	0.69	[0.44, 1.07]	1.02	[0.62, 1.69]
Health	0.93	[0.64, 1.35]	1.21	[0.78, 1.88]
Socially-related	0.73*	[0.52, 1.04]	0.66**	[0.47, 0.94]
**B: Self-Reported Mental Health**				
Caregiving Status	0.38***	[0.29, 0.49]	0.47***	[0.36, 0.63]
Caregiving Status* Male	3.23**	[1.36, 7.68]	1.83	[0.61, 5.49]
Caregiving Status* Male*Age	0.98**	[0.97, 1]	0.99	[0.97, 1.01]
Age	1.02***	[1.01, 1.03]	1.01**	[1, 1.02]
Male (ref. = female)	0.76*	[0.58, 0.99]	1.06	[0.76, 1.49]
Marital Status (ref. = married/common-law)				
Formerly married	0.57***	[0.43, 0.75]	0.64***	[0.48, 0.87]
Single	0.77**	[0.6, 0.98]	0.97	[0.69, 1.37]
Hours Worked	1	[0.99, 1.01]	1.01**	[1, 1.02]
Immigrant (ref. = Canadian born)	1.69***	[1.3, 2.2]	1.24	[0.9, 1.72]
Urban (ref. = rural)	0.77*	[0.58, 1.03]	0.81	[0.6, 1.1]
Education (ref. = less than high school)				
Trades, college, CEGEP, and other	0.94	[0.74, 1.19]	0.98	[0.74, 1.29]
Bachelor’s degree or lower university degree	0.99	[0.75, 1.3]	1.08	[0.77, 1.52]
University degree above bachelor’s	0.98	[0.7, 1.39]	1.36	[0.83, 2.24]
Income Groups (ref. = $100 K +)				
< $40 K	0.54***	[0.4, 0.72]	0.38***	[0.26, 0.55]
$40–$60 K	0.78*	[0.59, 1.04]	0.55***	[0.39, 0.79]
$60–$80 K	0.89	[0.66, 1.21]	0.76	[0.51, 1.12]
$80–$100 K	1	[0.69, 1.44]	0.8	[0.54, 1.19]
Occupation				
Management	1.06	[0.72, 1.56]	1.23	[0.71, 2.11]
Business	0.7**	[0.53, 0.91]	0.91	[0.64, 1.29]
Hard sciences	0.99	[0.65, 1.51]	0.93	[0.53, 1.63]
Health	1.03	[0.71, 1.5]	1.2	[0.69, 2.1]
Socially-related	1.08	[0.75, 1.55]	0.87	[0.56, 1.35]

Bold represents significant difference at 5% level between 2012 and 2018. *p < 0.1; **p < 0.05; ***p < 0.01

Caregiving was significantly associated with medium (GSS 2012: RRR = 1.35; GSS 2018: RRR = 1.54) or high (GSS 2012: OR = 1.59; GSS 2018: RRR = 1.66) as compared with low daily stress (Table 7). Additionally, male CEs were less likely to have medium daily stress than female CEs (GSS 2012: RRR = 0.51; GSS 2018: RRR = 0.30) and the magnitude would increase with age (GSS 2012: RRR = 1.01; GSS 2018: RRR = 1.02).

**Table 7:** Multinomial logistic models on daily stress, difficulty balancing job/home life, and work-life balance satisfaction, GSS cycles 2012 and 2018

	GSS cycle 2018	GSS cycle 2012	GSS cycle 2018	GSS cycle 2012
	Relative risk ratio	Relative risk ratio	Relative risk ratio	Relative risk ratio
**A: Daily Stress**	**Medium relative to low stress**		**High relative to low stress**	
Caregiving Status	1.54***	1.35***	1.66***	1.59***
Caregiving Status* Male	0.3**	0.51**	0.45	0.58
Caregiving Status* Male*Age	1.02***	1.01*	1.01	1.01
Age	0.99***	0.99**	0.99***	0.99**
Male (ref. = female)	0.85*	1	0.87	0.79**
Marital Status (ref. = married/common-law)				
Formerly married	1.1	1.08	1.33**	1.41***
Single	0.78**	0.86*	0.78**	0.73***
Hours Worked	1.01***	1.01***	1.02***	1.03***
Immigrant (ref. = Canadian born)	1.13	1.02	0.93	1.04
Urban (ref. = rural)	1.02	1.19**	1.42***	1.2*
Education (ref. = less than high school)				
Trades, college, CEGEP, and other	1.16	0.96	1.22*	1.18*
Bachelor’s degree or lower university degree	1.11	1.27**	1.64***	1.43***
University degree above bachelor’s	1.39**	1.35**	1.86***	1.67***
Income Groups (ref. = $100 K +)				
< $40 K	1.14	1.1	1.48**	1.4**
$40–$60 K	0.9	0.97	1.03	0.95
$60–$80 K	1.13	1.02	1.17	0.9
$80–$100 K	0.96	0.99	0.98	0.94
Occupation				
Management	1.32*	1.99***	1.68***	2.36***
Business	0.94	1.16	0.91	0.91
Hard sciences	0.95	1.18	0.85	0.87
Health	1.05	1.48**	0.93	1.09
Socially-related	0.86	1.04	0.81	0.66**
**B: Difficulty Balancing Job/Home Life**	**Medium relative to low difficulty**		**High relative to low difficulty**	
Caregiving Status	2.22***	1.8***	2.52***	1.34
Caregiving Status* Male	0.46**	1.24	0.2**	0.39
Caregiving Status* Male*Age	1.01	0.99	1.03*	1.02
Age	0.99***	0.98***	0.99*	0.97***
Male (ref. = female)	0.87*	0.79***	0.71	0.65
Marital Status (ref. = married/common-law)				
Formerly married	1.09	0.94	1.12	1.12
Single	0.45***	0.44***	0.49**	0.21***
Hours Worked	1.02***	1.03***	1.04***	1.04***
Immigrant (ref. = Canadian born)	1.36***	1.26***	1.82**	1.9**
Urban (ref. = rural)	1.17*	0.92	1.48	1.3
Education (ref. = less than high school)				
Trades, college, CEGEP, and other	1.19*	1.33***	1.02	0.89
Bachelor’s degree or lower university degree	1.36***	1.32***	1.23	0.92
University degree above bachelor’s	1.52***	1.54***	0.66	0.83
Income Groups (ref. = $100 K +)				
< $40 K	1.2	1.47***	1.35	4.02***
$40–$60 K	1.04	1.16*	2.36***	2.94***
$60–$80 K	1.09	1.06	1.49	0.95
$80–$100 K	1.19	1.06	1.19	1.02
Occupation				
Management	1.05	0.92	1.68*	2.41**
Business	0.88	0.76***	0.66	1.19
Hard sciences	0.8	0.75**	0.58	0.55
Health	1.07	1.03	0.84	1.3
Socially-related	0.99	0.76**	1.24	0.93
**C: Work-life Balance Satisfaction**	**Indifferent relative to satisfied**		**Dissatisfied relative to satisfied**	
Caregiving Status	1.34***	1.43***	1.54***	1.43***
Caregiving Status*Immigrant	1.17	0.96	1.84**	0.93
Age	0.99***	0.98***	0.99***	0.99***
Male (ref. = female)	0.92	0.82**	0.86	0.75**
Marital Status (ref. = married/common-law)				
Formerly married	1.12	1.09	1.29*	1.52***
Single	0.85	0.98	0.6***	0.84
Hours Worked	1.01***	1.02***	1.03***	1.02***
Immigrant (ref. = Canadian born)	0.99	0.97	0.7**	0.98
Urban (ref. = rural)	1.45***	1.29**	1.56***	1.28**
Education (ref. = less than high school)				
Trades, college, CEGEP, and other	1.04	1.31**	1.29*	1.12
Bachelor’s degree or lower university degree	1.13	1.59***	1.71***	1.18
University degree above bachelor’s	1.13	1.42**	1.94***	1.01
Income Groups (ref. = $100 K +)				
< $40 K	1.1	1.33**	1.63***	1.78***
$40–$60 K	0.99	1.29**	1.21	1.36*
$60–$80 K	1.14	1.11	1.31*	0.86
$80–$100 K	1.17	1.19	1.06	1.18
Occupation				
Management	1.46**	1.14	1.02	1.08
Business	1.31**	1.14	1.09	0.98
Hard sciences	1.07	1.05	0.9	0.99
Health	1.28	1.18	1.2	0.96
Socially-related	1.35**	0.87	0.86	0.86

Bold represents significant difference at 5% level between 2012 and 2018; *p < 0.1; **p < 0.05; ***p < 0.01

Caregiving was significantly related to medium difficulty balancing job/home life vs. low difficulty (GSS 2012: RRR = 1.80; GSS 2018: RRR = 2.22; Table 7). In 2012, caregiving was also significantly associated (*p* < 0.01) with high difficulty balancing job/home life (GSS 2012: RRR = 2.52) but not in 2018. In addition, male CEs were less likely to have medium or high (GSS 2018: RRR = 0.46 or 0.20) difficulty in 2018 but not significant in 2012.

Caregiving has significant association with indifference or dissatisfaction with work-life balance vs. satisfaction (GSS 2012: RRR = 1.43 or 1.43, respectively; GSS 2018: RRR = 1.34 or 1.54; Table 7), as well as the lower levels of life satisfaction (Table 8) and the magnitude increases with time.

**Table 8:** OLS linear regression on life satisfaction, GSS cycles 2012 and 2018

Life satisfaction	GSS cycle 2018		GSS cycle 2012	
	Coefficient	95% CI	Coefficient	95% CI
Caregiving Status	-0.421***	**[-0.54, -0.302]**	-0.22***	**[-0.297, -0.143]**
Age	-0.004**	[-0.008, 0]	-0.001	[-0.003, 0.003]
Male (ref. = female)	0.042	[-0.079, 0.163]	0.013	[-0.069, 0.095]
Marital Status (ref. = married/common-law)				
Formerly married	-0.306***	[-0.459, -0.154]	-0.493***	[-0.626, -0.361]
Single	-0.376***	[-0.522, -0.23]	-0.281***	[-0.391, -0.17]
Hours Worked	-0.003*	[-0.007, 0.001]	-0.003*	[-0.006, 0]
Immigrant (ref. = Canadian born)	-0.01	[-0.141, 0.12]	-0.21***	[-0.31, -0.11]
Urban (ref. = rural)	-0.402***	[-0.532, -0.273]	-0.291***	[-0.383, -0.2]
Education (ref. = less than high school)				
Trades, college, CEGEP, and other	-0.123*	[-0.259, 0.014]	-0.033	[-0.131, 0.065]
Bachelor’s degree or lower university degree	-0.259***	[-0.412, -0.106]	-0.015	[-0.117, 0.087]
University degree above bachelor’s	-0.214**	[-0.394, -0.034]	-0.127*	[-0.265, 0.012]
Income Groups (ref. = $100 K +)				
< $40 K	-0.639***	[-0.823, -0.454]	-0.585***	[-0.722, -0.448]
$40–$60 K	-0.389***	[-0.558, -0.22]	-0.333***	[-0.454, -0.213]
$60–$80 K	-0.312***	[-0.479, -0.146]	-0.231***	[-0.336, -0.126]
$80–$100 K	-0.224***	[-0.387, -0.062]	-0.179***	[-0.288, -0.07]
Occupation				
Management	0.088	[-0.096, 0.271]	-0.035	[-0.177, 0.107]
Business	-0.2***	[-0.349, -0.051]	-0.074	[-0.18, 0.033]
Hard sciences	-0.186*	[-0.391, 0.018]	0.012	[-0.131, 0.156]
Health	-0.044	[-0.255, 0.166]	-0.041	[-0.199, 0.116]
Socially-related	-0.13	[-0.318, 0.059]	-0.049	[-0.185, 0.087]

Bold represents significant difference at 5% level between 2012 and 2018; *p < 0.1; **p < 0.05; ***p < 0.01

### Linear and multinomial logistic on intensity of caring

In Table 9, female CEs provided 4.2 h more care than male CEs in 2018 on average, but only 1.9 h more in 2012. Specifically, using no intensive care as base outcome, male CEs were less likely than female CEs to provide transportation at least once a week in 2012 but not significant in 2018. Male CEs were less likely than female CEs to provide help daily or at least once a week with household chores, medical treatment or scheduling in both surveys, but there is no significant change over time. Male CEs were more likely than female CEs to provide help for house maintenance at least once a week in both surveys with similar magnitudes.

**Table 9:** Linear regression on the number of hours of caring and multinomial logistic models on the frequency of caring tasks

	Number of hours (per week)		Transportation				Household chores			
			At least once a week vs. no intensive care		Daily care vs. no intensive care		At least once a week vs. no intensive care		Daily care vs. no intensive care	
	Coeff	Coeff	Relative risk ratio		Relative risk ratio		Relative risk ratio		Relative risk ratio	
	2018	2012	2018	2012	2018	2012	2018	2012	2018	2012
Male	-4.2***	-1.9***	1.14	0.74***	1.1	0.73	0.78*	0.65***	0.65***	0.66***
Age	0.18***	0.1***	1.03***	1.02***	1.03***	1.02**	1	0.99*	1.02***	1.01**
Marital Status (ref. = married/common-law)										
Formerly married	*-1.99*	4.08**	1.42**	0.84	0.85	1.46	1.68***	1.28	0.91	0.99
Single	*1.17*	*0.47*	1.27	1.3*	1.09	1.29	2***	1.23	1.5*	1.81***
Hours Worked	0.6	0.77	1.63***	1.29*	3.04	2.5***	1.15	1.19	2.47	2.46***
Immigrant (ref. = Canadian born)	0.09*	0.01	1	1.01***	1	1.01**	1.01	1	1	1
Urban (ref. = rural)	1.66	-0.31	1.23	1.26**	1.19	1.76**	1.04	1.03	1.22	1.22
Education (ref. = less than high school)										
Trades, college, CEGEP, and other	*-2.1*	-1.09*	0.98	0.98	0.87	0.57**	0.89	0.99	0.77	0.71**
Bachelor’s degree or lower university degree	-5.46***	-2.44***	0.88	0.95	0.5**	0.97	1.03	1.1	0.56***	0.58***
University degree above bachelor’s	-6.46***	*-0.81*	0.7*	0.85	0.52**	0.51	0.93	1.08	0.41***	0.64*
Income Groups (ref. = $100 K +)										
< $40 K	*2.37*	3.57***	0.89	1.28	1.6	1.99**	0.92	1.13	1.68**	2.54***
$40–$60 K	9.63***	2.24**	1.46**	1.2	2.71***	1.39	1.07	1.67***	1.61**	1.45*
$60–$80 K	2.31*	*0.56*	0.9	1.21	1.61	1.5	0.9	1.3	1.28	1.44*
$80–$100 K	*1.02*	*0.11*	0.73*	1.26*	1.63	1.51	0.88	1.15	0.89	1.38
	House maintenance				Medical treatments					
	At least once a week vs. no intensive care		Daily care vs. no intensive care		At least once a week vs. no intensive care		Daily care vs. no intensive care			
	2018	2012	2018	2012	2018	2012	2018	2012		
Male	1.92***	1.79***	1.21	1.72**	0.67**	0.66**	0.64**	0.68**		
Age	1	1	1.02**	1.02*	1	1	1.04***	1.03***		
Marital Status (ref. = married/common-law)										
Formerly married	0.99	0.95	0.75	1.25	1.31	1.09	0.52*	1.24		
Single	1.36	1.39**	0.85	2.95***	0.99	0.72	0.88	2.07***		
Hours Worked	0.94	1.03	0.99	2.14**	2.1***	1.35	2.03	1.92**		
Immigrant (ref. = Canadian born)	1	1	1.01**	1.01	1	1	1.01***	1.01**		
Urban (ref. = rural)	0.91	0.87	1.05	1.08	0.99	1.34	0.79	1.46		
Education (ref. = less than high school)										
Trades, college, CEGEP, and other	1.11	0.82	0.82	0.61**	0.89	1.23	0.8	1.05		
Bachelor’s degree or lower university degree	0.87	0.69**	0.47***	0.51**	0.68	1	0.56**	0.39***		
University degree above bachelor’s	0.63*	0.44***	0.33***	0.67	0.73	1.29	0.37**	0.93		
Income Groups (ref. = $100 K +)										
< $40 K	1.09	1.18	3.18***	2.1**	1.03	1.61*	2.23**	2.37***		
$40–$60 K	1.23	1.33*	1.94*	1.52	0.89	1.11	1.92*	1.7*		
$60–$80 K	1.04	1.66***	1.95**	1.13	1.54	1.26	2.06**	1.02		
$80–$100 K	0.81	1.46**	1.84**	1.86**	0.79	0.89	1.75	1.44		
	Scheduling				Banking					
	At least once a week vs. no intensive care		Daily care vs. no intensive care		At least once a week vs. no intensive care		Daily care vs. no intensive care			
	2018	2012	2018	2012	2018	2012	2018	2012		
Male	0.62***	0.66***	0.66*	0.55*	0.71**	0.8	0.73	0.78		
Age	1.03***	1.02***	1.06***	1.05***	1.06***	1.04***	1.04***	1.05***		
Marital Status (ref. = married/common-law)										
Formerly married	0.96	1.25	1.14	1.1	0.55**	0.87	0.34**	0.51		
Single	1.1	0.67	1.19	0.63	0.64*	0.8	1.46	0.71		
Hours Worked	2.21***	1.19	1.93	1.36	1.15	0.9	1.71	0.94		
Immigrant (ref. = Canadian born)	1	1	1	1.01	1	1.01	1	1.03***		
Urban (ref. = rural)	1.21	1.19	0.98	1.2	1.44**	1.09	1.25	1.31		
Education (ref. = less than high school)										
Trades, college, CEGEP, and other	0.81	1.21	1.18	0.9	0.99	1	1.51	1.18		
Bachelor’s degree or lower university degree	0.81	1.04	0.58	1.83	1.28	1.1	1.1	1.13		
University degree above bachelor’s	0.82	1	1.11	1.79	0.91	1.37	1.57	0.79		
Income Groups (ref. = $100 K +)										
< $40 K	0.89	0.97	1.44	2.99*	1.23	1.62**	0.5	3.88**		
$40–$60 K	1.01	1.05	2.33**	1.36	1.36	1.31	1.64	2.11		
$60–$80 K	1.15	1.14	1.82*	1.52	1.08	1.49**	1.15	2.08		
$80–$100 K	0.89	1	0.84	1.32	1.37	1.64**	0.76	2.89**		

Bold represents significant difference at 5% level between 2012 and 2018; *p < 0.1; **p < 0.05; ***p < 0.01

## Discussion

This paper identifies trends in the socio-demographic, health, and work profile of Canadian carer-employees (CEs) over time via the analysis of the 2012 and 2018 cycles of Canada’s General Social Survey. We found that in comparison with non-CEs, CEs were more likely to have: medium to high daily stress; medium difficulty balancing job/home life; indifference or dissatisfaction with work-life balance; poor general and mental health; and lower levels of life satisfaction. These findings are consistent with prior research showing CEs find caregiving to be highly stressful and emotionally draining, and are more likely to exhibit psychological distress and unfavourable moods (Sherman, 2018; Sethi et al., 2017; Robison et al., 2009). Our study also indicates female CEs had increased association in daily stress and difficulty balancing job/home life compared to the male CEs. According to the latest release from Statistics Canada (https://www150.statcan.gc.ca/n1/daily-quotidien/221108/t004b-eng.htm), the impacts were greater for women across several different health-related symptoms, such as feeling anxious, overwhelmed, short-tempered or irritable, or depressed.

Additionally, general health and life satisfaction for CEs were significantly worsened from 2012 to 2018. This may be due to work intensification, characterized by work increasing in complexity, becoming faster paced, and requiring different tasks to be done concurrently (Paškvan & Kubicek, 2017). Recent research has shown the effect of work intensification on employee well-being is mostly negative (Paškvan & Kubicek, 2017), is associated with reduced work-life balance (Macky and Boxall 2008) and reduced employee satisfaction (Brown, 2012), and leads to heightened emotional exhaustion (Korunka et al., 2015). Our research indicates that CEs in 2018 are more likely to have higher difficulties with work due to family responsibilities rather than the converse, which may mean they are disproportionately affected by work intensification.

Another key finding is that males and females were equally likely to be CEs in 2018 but women were marginally overrepresented among CEs in 2012. While a growing proportion of male CEs has been observed in the existing North American literature (Fast et al., 2014), this result shows evidence that among Canadian CEs, the gender gap has effectively disappeared. It was estimated that 80% of unpaid care was provided by women in 2004 (Grant et al., 2004). The dramatic demographic shift we observe since then can be attributed to a number of factors. First, in North America, social attitudes towards gender roles have steadily become more egalitarian (Donnelly et al., 2016). Among men, childcare roles are becoming more accepted, which may parallel an acceptance of eldercare roles. These shifting social attitudes may contribute to a willingness for male employees to take on a caring role for their elderly relatives. Second, the shift towards smaller family sizes may put men in a position where only they are able to take care of their aging parents. According to Statistics Canada (2018b), the average household size has been below 3 since the mid-1970s. Regardless, our findings suggest greater carer-friendly workplace supports need to be integrated into male-dominated labour sectors, such as construction, transport, and resource extraction. Despite this, gender disparities still exist due to differences in the nature of caregiving. Consistent with the findings by Maynard et al. (2018), this study also found that male CEs typically spend fewer hours per week providing care and are less likely to engage in intensive care (Maynard et al., 2018). In addition, this gender gap would increase over time in Canada.

The study provides the evidence that CEs’ health and well-being have worsened over time, especially for female CEs, indicating that the needs of CEs are growing at a faster rate than the supports available. Our findings are useful in informing and justifying the provision of CE supports at work in order to sustain CEs in the workplace. This is particularly important given that CEs are often the most experienced workforce, given that they are primarily between the ages of 45 and 65 years. Losing CEs from the labour market will add to the recruitment, retention, and turnover challenges that employers are experiencing, and only further complicate the shortages being experienced in the labour market. In addition, replacing an employee is expensive, as noted in a study by the Society of Human Resource Management which indicated it costs up to nine months of an employee’s salary to find and train their replacement (Lynchburg Regional SHRM, 2017). As the prevalence of CEs and the stresses associated with caregiving increase over time, it is incumbent upon employers to support CEs in the workplace through the creation of carer-friendly workplace policies (CFWPs) (Mofidi et al., 2019). CFWPs are wide-ranging and mainly include: educational workshops and counselling; flexible and customizable work schedules; financial assistance or relief; unpaid leave; paid leave; and changes to workplace culture (Ireson et al., 2018). McMaster University (Hamilton, Ontario, Canada) partnered with the Canadian Standards Association to create a voluntary carer-standard, entitled *CSA B701-17 Carer-inclusive and accommodating organizations standard* (Canadian Standards Association Group, 2017). Further, an implementation handbook, entitled *CSA B701HB-18 Helping worker-carers in your organization* (Canadian Standards Association Group, 2018), was built to complement the Standard. Both the Standard and handbook are currently available as complementary downloads.

In the past, it has been suggested that CFWPs be targeted towards female-dominated labour sectors, such as education, business, sales, and service, due to their overrepresentation among CEs (Wang et al., 2018). Current data indicate that attention to male-dominated labour sectors is now needed and presents a new sphere of intervention science research. Additionally, while immigrants are still less likely to be CEs than non-immigrants, they are much more likely to be CEs in 2018 than in 2012. This trend warrants further research. The worsening health outcomes and shifting demographics of CEs have clear implications for human resource management, public health, and occupational health and safety.

### Strengths and limitations of the study

There are several immediate limitations to our analysis. First, as with all secondary data, variables of interest are not always present. Second, cross-sectional analysis cannot be used to make causal inferences, meaning we cannot confirm the presumed effects of caregiving on health, well-being, and work. Third, questions from the 2012 and 2018 GSS cycles occasionally were not identical or had different options for response. A prime example would be the responses related to work-life balance, which used a 4-point scale in 2012 and a 5-point scale in 2018. In this case, the analysis required categories to be collapsed to align responses from 2018 and 2012, which may have introduced bias.

## Conclusion

This paper updated the profile of carer-employees by comparing two cycles of Canada’s GSS: while women were more likely to be CEs in 2012, the gender difference was insignificant in 2018. The outcomes regarding the health, well-being, work and intensity of caring have either stayed consistent or worsened with respect to the role of CE from 2012 to 2018 and the associations were greater for female CEs, providing evidence that the needs of CEs are growing at a faster rate than the supports available. The results are meaningful in informing and justifying the provision of CE supports at work in order to sustain CEs in the workplace, such as the carer-friendly workplace policies.

## Contributions to knowledge

What does this study add to existing knowledge?

- While women were more likely to be CEs in 2012, the gender difference was insignificant in 2018.
- The intensity care for female CEs as compared with their male counterparts was significantly increased from 2012 to 2018.
- Outcomes regarding health, well-being, and work have either stayed consistent or worsened for CEs between 2012 and 2018.
- The needs of Canadian CEs are likely growing at a faster rate than the supports available.

What are the key implications for public health interventions, practice or policy?

- The results are useful in informing and justifying the provision of CE supports at work in order to sustain CEs in the workplace.
- One novel strategy for CEs in the workplace is the creation of carer-friendly workplace programs (CFWPs).
- CFWPs include a range of workplace initiatives, such as carer support groups, education and training seminars, peer-support programs, specialized training for supervisors/managers, as well as interventions to support a carer-friendly workplace culture.

## Data Availability

Data access is through Statistics Canada Research Data Centre (RDC) at McMaster University. Access to data is restricted and the data cannot be released outside of the RDC due to confidentiality issues.
